# 3D Anodic Alumina Nanoarchitectures: A Decade of Progress from Foundational Science to Functional Metamaterials

**DOI:** 10.1002/adma.202514821

**Published:** 2025-11-22

**Authors:** Marisol Martín‐González

**Affiliations:** ^1^ CSIC (CEI UAM+CSIC) Instituto de Micro y Nanotecnología (IMN‐CNM) Isaac Newton, 8 Tres Cantos Madrid 28760 Spain

**Keywords:** 3D anodic alumina (3D‐AAO), all‐day radiative cooling, interconnected nanonetworks, metamaterials, triboelectric generators

## Abstract

Three‐dimensional (3D) nanostructures are redefining materials engineering by shifting control from composition to architecture. This review charts the rise of 3D anodic aluminum oxide (3D‐AAO), beginning with the 2014 introduction of ordered, interconnected nanoarchitectures that established a new paradigm. We detail fabrication advances that enable precise control of geometry, pore shape, and periodicity, including hybrid pulse anodization that alternates between hard anodization (HA) and mild anodization (MA) conditions. These architected templates used empty or as scaffolds for magnetic, semiconducting, and polymeric phases act as metamaterials with tunable properties. In magnetism, geometry enables easy‐axis reversal and uncovers magnetoelastic anisotropy. In semiconductors, 3D nanonetworks raise the dimensionless thermoelectric figure of merit (zT) relative to similarly prepared bulk via geometry‐driven phonon scattering. In polymers, 3D‐AAO guides flexible photonic devices with full‐gamut structural color and biodegradable, biocompatible triboelectric nanogenerators with high power density. Empty 3D‐AAO offers passive daytime radiative cooling properties. Delivering 〉10 °C sub‐ambient cooling, passively reduces building air‐conditioning demand. The field's influence spans energy storage, catalysis, and biosensing. We conclude with pathways to industrial scalability through cost‐efficient, room‐temperature processing on low‐purity aluminum, positioning 3D‐AAO to bridge nanoscale physics and macroscale function.

## Graphical abstract

Ordered three‐dimensional anodic aluminum oxide (3D‐AAO) nanoarchitectures with longitudinal and transverse pores enable architecture‐driven metamaterials. The review maps fabrication advances, including hybrid pulse anodization, and shows how 3D‐AAO templates tailor properties across magnetism, energy, catalysis, and sensing. Intrinsic 3D‐AAO also offers passive radiative cooling and ultralow thermal conductivity, bridging nanoscale design and macroscale function.

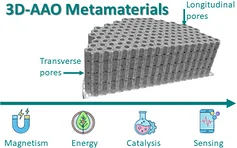

## 1 Introduction: A Paradigm Shift from 1D to 3D Nanotemplates

The continuous drive for miniaturization and advanced functionality has propelled materials science into the nanoscale realm. For several decades, 1D nanostructures, such as nanowires and nanotubes,^[^ ^1^ ^]^ along with their 2D arrays, have been the subject of intensive research. While these systems have been invaluable in unveiling a wealth of nanoscale physical phenomena and applications, their inherent dimensional constraints often limit transport properties (e.g., phonon/electron mean free path, ionic diffusion) and interfacial interactions. Consequently, the subsequent logical frontier involved the transition into the third dimension, aiming to create complex, interconnected networks capable of exhibiting tunable anisotropy, enhanced mechanical stability, and novel collective phenomena, while also providing a practical route to translate nanoscale effects into robust, handleable macroscale devices for real‐world applications.

Unlike other emerging 3D nanofabrication approaches such as nanoscale additive manufacturing,^[^ ^2^ ^]^ DNA‐origami scaffolds,^[^ ^3^ ^]^ or metal nanowire aerogels,^[^ ^4^ ^]^ nanoporous Anodic Aluminum Oxide (AAO)^[^ ^5^ ^]^ – also known as Anodic Porous Alumina (APA), Nanoporous Anodic Alumina (NAA), Porous Anodic Alumina (PAA), Porous Aluminum Oxide (PAO), or Nanoporous Anodized Alumina Platforms (NAAP) – combines scalability, cost‐effectiveness, and structural regularity, positioning it uniquely for engineering programmable 3D nanoarchitectures. A comparative table showing the 3D‐AAO versus other nanofabrication approaches can be observed in **Table** **1**.

**Table 1:** Technology comparison table.

Technology	Resolution [nm]	Scalability	Cost	Processing Time
3D‐AAO	≈10–500	High	Low	Hours‐Days
Additive Manufacturing	≈50–1000	Medium‐High	Medium	Hours‐Weeks
DNA Origami	≈1–50	Low‐Medium	Medium	Days
Metal Aerogels	≈100–1000	Medium	Medium	Hours

A pivotal milestone in this field of AAO was the work in 2014,^[^ ^6^ ^]^ which, for the first time, demonstrated the fabrication of ordered 3D interconnected nanoarchitectures within anodic porous alumina. By developing a pulsed anodization technique followed by controlled selective chemical etching, it became possible to create periodic planes of transverse nanochannels that interconnect the primary longitudinal pores. This process transformed the conventional AAO template from a series of isolated columns into a continuous 3D‐AAO nanoscaffold and nanonetwork. This breakthrough constituted more than a mere incremental advance; it represented a significant technological advance, establishing a programmable platform where the geometry itself—specifically, the interconnection distance, channel diameter, and periodicity—could be employed as a primary tool to design and dictate a material's macroscopic properties. Crucially, these 3D‐AAO structures are composed of a hexagonal array of straight, vertical nanopores and periodic transverse nanochannels that interconnect neighboring pores in well‐defined planes, yielding a defect‐free, fully interconnected tubular network in all three spatial directions. This precise architecture, obtained by alternating mild‐ and hard‐anodization pulses under current‐limited conditions, allows for independent tuning of interconnection periodicity (P), pore diameter, and overall thickness, while preserving macroscopic uniformity and plane‐by‐plane interconnectivity. This innovation thus provided a novel approach to designing metamaterials at the nanoscale and a means to translate nanoscale properties to the macroscale.

By contrast, several architectures in the literature are sometimes labelled as “3D‐AAO” but differ fundamentally from the definition used here; to avoid ambiguity, we reserve the term 3D‐AAO for the periodic transverse channels forming plane‑by‑plane interconnectivity in the 3 dimensions as described above and refer to other systems by the more precise descriptors “branched AAO”, “hierarchical AAO”, or “diameter‐modulated AAO”.

## 2 Engineering 3D Anodic Alumina Nanoarchitectures

The precise control over its complex architecture is fundamental to harnessing the full potential of 3D‐AAO. The evolution from 1D pores arrays to intricate 3D networks has been primarily driven by advancements in electrochemical engineering.

### 2.1 3D Interconnected Nanonetworks Synthesis

**Figure 1:**
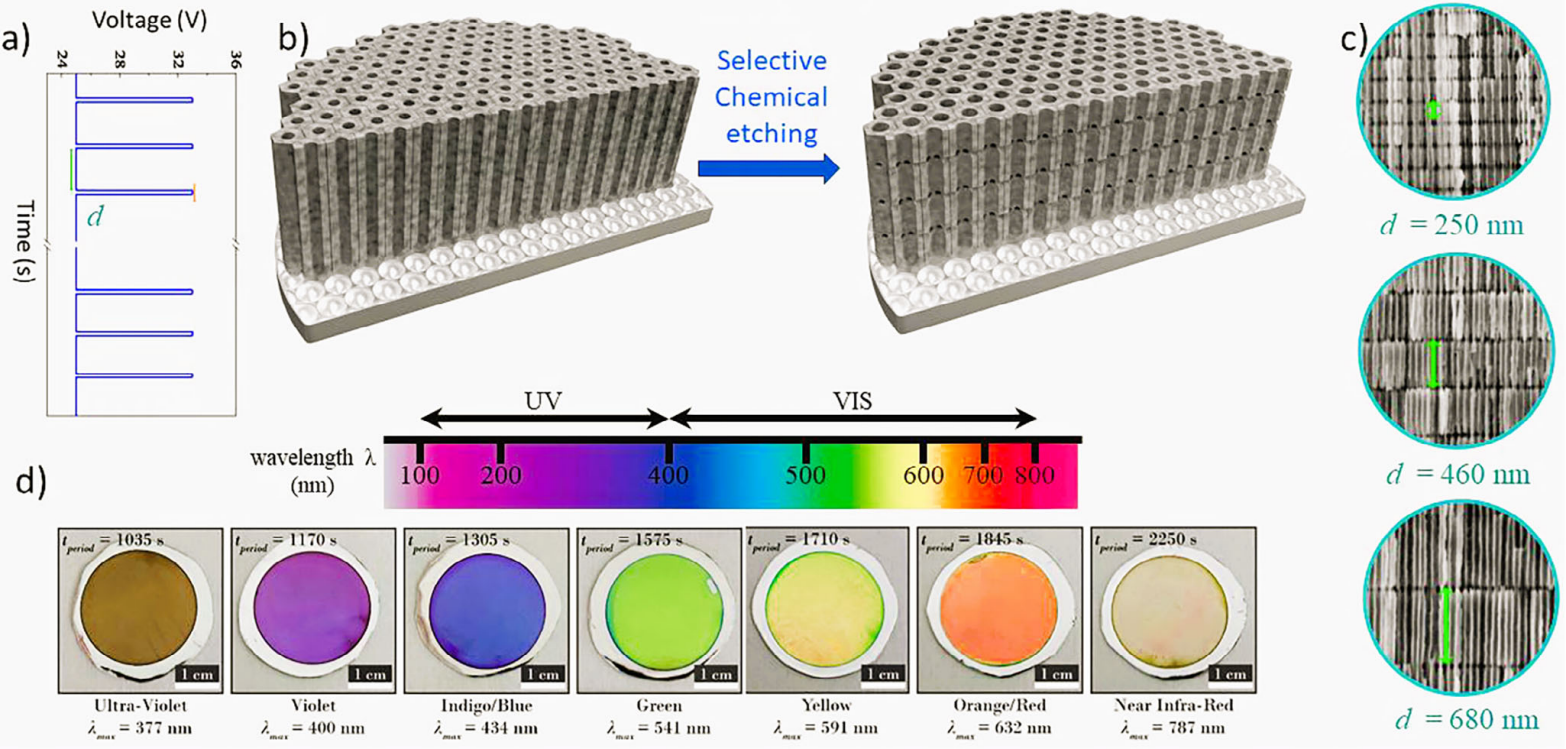
Schematic overview of the fabrication and tunable Bragg refractor properties of 3D nanoporous anodic alumina (3D‐AAO) structures. a) llustrates pulse anodization, where voltage or current pulses program the periodicity of 3D‐AAO, which after selective chemical etching, this encoded modulation is exposed, producing the 3D interconnected network that underpins the tunable structural color. b) 3D renderings of 3D‐AAO before and after selective chemical etching, illustrating the creation and refinement of interconnected nanoporous architectures. c) Cross‐sectional SEM images (up to down) show representative 3D‐AAO templates with controlled interconnected channel distances of 250 , 460 , and 680 nm, achieved via adjustment of anodization time under mild anodization conditions (MA). d) Optical photographs of 3D‐AAO membranes displaying vivid, tunable structural coloration across the visible spectrum and into the ultraviolet (λ_max_ = 377 nm) and near‐infrared (λ_max_ = 787 nm) regions, as a function of pulse duration. These results demonstrate that photonic stop‐band position and full‐gamut, pigment‐free color response can be programmed through nanoscale control of 3D‐AAO architecture (the figure has been adapted from refs. [9, 10, 11]).

The original methodology enables control over interconnection periodicity (P) in the range of ≈150–800 nm,^[^ ^6^ ^]^ with pore diameters of 40–65 nm and interpore distances of 65 nm,^[^ ^6^ ^]^ as demonstrated in the foundational work. This control is obtained by pulsing the voltage or the current during anodization‐ periodically alternating between mild anodization (lower voltage) and hard anodization (higher voltage) conditions or lower current and higher current‐ and by adjusting the time of pulses under mild anodization conditions. The oxide layers formed during the HA phase, being more chemically reactive, are then preferentially dissolved by a subsequent chemical etching step. Chemical etching is typically performed using 5 wt.% H_3_PO_4_ solution at room temperature for controlled times (1–30 min), which preferentially dissolves the alumina formed during hard anodization steps while dissolving more slowly the oxide formed during mild anodization conditions.^[^ ^6^ ^]^ This generates a network of transverse nanochannels that interconnect the longitudinal pores formed during the MA steps. The shape of transverse nanochannels can be tuned by using different pulse waveforms such as square, sinusoidal, or sawtooth. For the square pulse waveforms, transverse nanochannels present an oval shape with dimensions of ≈20 nm (short diameter) to ≈35 nm (long diameter) depending on etching conditions.^[^ ^6^, ^7^ ^]^ This provides benchmarking against other 3D material synthesis approaches in terms of achievable structural parameters,^[^ ^8^ ^]^ see **Figure** **1**.

Since this initial breakthrough, further refinements have afforded even greater command over the 3D geometry. By modulating not only the voltage but also the waveform of the electrochemical signal (e.g., square, sinusoidal, sawtooth), it is possible to fine‐tune the morphology of the transverse pores, including their width and height.^[^ ^5^, ^7^ ^]^ This, in turn, influences the local porosity and introduces an additional degree of freedom for engineering the template's properties.^[^ ^5^, ^7^ ^]^ Square waveforms produce transverse nanochannels with sharper pore diameter transitions (±5 nm variation), sinusoidal waveforms create gradual transitions with enhanced optical properties (noticeably improved color purity), while sawtooth waveforms enable asymmetric pore profiles beneficial for directional transport applications.^[^ ^7^ ^]^ Recent advancements have also demonstrated the successful synthesis of 3D‐AAO architectures in lower‐purity (and thus cheaper) aluminum foils, such as aeronautical‐grade aluminum (i.e., AA2024‐T3, which is an Al–Cu–Mg alloy with typical composition Cu 3.8%–4.9%, Mg 1.2%–1.8%, Mn 0.3%–0.9%, with Al content typically ranging from 90.7% to 94.7% by weight), highlighting the material's excellent intrinsic properties and expanding its potential applications where material purity is critical. This approach further broadens the scope of 3D‐AAO fabrication techniques by reducing the price of the aluminum used.^[^ ^9^, ^12^ ^]^ Alternative synthesis routes have also been investigated, such as the anodization of aluminum alloys. For instance, in Al–Cu alloys, local compositional inhomogeneities—such as Cu‐rich regions at the metal/oxide interface—can bias the anodization process, occasionally promoting lateral pore formation without the need of voltage pulsing. This effect arises from alloy‐assisted variations in oxide growth rate rather than a programmed pulsing protocol.^[^ ^13^ ^]^ A more recent work has also demonstrated the successful synthesis of conformal 3D porous anodic alumina networks on cylindrical aluminum wires, thereby illustrating the versatility of this fabrication method for non‐planar geometries.^[^ ^14^ ^]^

It is important to say that classical approaches already used in AAOs like two‐step anodization method^[^ ^8^ ^]^ or pre‐patterning aluminum surfaces^[^ ^15^ ^]^ can also be used to order the longitudinal pores before the 3D‐AAO anodization step. In this particular case, an initial anodization on high‐purity aluminum at the constant voltage of the MA has to be used, followed by selective chemical dissolution of the first oxide layer to create a pre‐patterned surface. The subsequent second anodization step utilizes the same conditions as the first, but the pre‐patterned surface ensures highly ordered pore nucleation and growth.^[^ ^5^ ^]^ This approach can be extended to multiple cycles, with some researchers developing methods to produce multiple AAO films from a single aluminum substrate through alternating anodization and cathodic delamination.^[^ ^16^ ^]^ Alternative approaches to the two‐step process include pre‐patterning aluminum surfaces^[^ ^15^ ^]^ using nanoimprint lithography or other texturing methods. These techniques can guide pore nucleation and growth, potentially reducing processing time while maintaining structural order.^[^ ^5^, ^8^, ^15^ ^]^

### 2.2 Intrinsic Properties of Empty 3D‐AAO Templates

Beyond their function as scaffolds, which will be reviewed in the subsequent section, the 3D‐AAO templates possess distinctive intrinsic properties that are valuable in their own right.

#### 2.2.1 Optical Properties

The periodic modulation of porosity along the pore axis renders 3D anodized aluminum oxide (3D‐AAO) a natural photonic crystal, specifically functioning as a Distributed Bragg Reflector (DBR) with inherent optical tunability,^[^ ^5^, ^6^, ^7^, ^9^, ^10^ ^]^ see Figure 1. Pioneering work established all‐dielectric 3D mesoporous network metamaterials through innovative anodization techniques, achieving a full gamut of structural colors by precisely controlling interconnection periodicity (P) and refractive index contrast via sinusoidal waveforms; this novelty enables broadband absorption, angle‐dependent iridescence, and scalable patterns for anti‐counterfeiting, marking a significant advance over traditional 2D photonic structures.^[^ ^10^ ^]^ Building on this, recent advancements incorporate diverse waveforms—such as sawtooth and square profiles—along with plasmonic metal layers (e.g., gold or silver) to create hybrid photonic‐plasmonic modes, yielding RGB‐specific colors with enhanced purity, reduced angle dependence, and 3D interconnectivity ideal for high‐resolution display technologies.^[^ ^5^, ^7^ ^]^ These structures exhibit vibrant, tunable structural coloration, where the primary hue is dictated by the Bragg condition and further refined by waveform adjustments that modulate layer contrasts.^[^ ^5^, ^6^, ^7^, ^10^ ^]^ Collectively, such optical functionalities have been harnessed for diverse applications, including real‐time sensing, anti‐counterfeiting, and next‐generation displays, underscoring the versatility of 3D‐AAO metamaterials in bridging fundamental photonics with practical innovations.^[^ ^10^ ^]^

It is important to highlight here that similar behavior can also be observed in modulated 1D nanoporous templates, which have been much more extensively studied in the literature but do not allow for the generation of 3D networks of nanowires, as it will be seen later. For instance, a review on modulated nanoporous anodic alumina (NAA) photonic crystals details their fabrication via anodization techniques, tunable optical properties (e.g., photonic stopbands and effective medium engineering), versatile surface modifications for selectivity, and applications in real‐time optical chemo‐ and biosensing, such as detecting metal ions, vitamins, and biomolecules through techniques like reflectometric interference spectroscopy.^[^ ^17^ ^]^ All of these functionalities can likewise be realized in 3D‐AAO platforms, with the added benefit of enabling interconnected 3D nanowire networks. This underscores AAO's advantages over traditional templates in enabling multidimensional photonic structures with high sensitivity and broad applicability.

#### 2.2.2 Thermal Conductivity and Management Properties

More recently, the thermal management properties of 3D‐AAO have revealed information about energy‐efficiency enhancements due to architecture. Characterization of the mid‐infrared (mid‐IR) optical properties of AAOs have revealed its potential for passive daytime radiative cooling. 3D‐AAO structures on aluminum can be engineered to possess high solar reflectance and high mid‐IR emissivity, enabling them to achieve temperatures exceeding 10 °C below ambient conditions under direct sunlight without requiring electrical power.^[^ ^18^ ^]^ This transformative advance has been achieved by demonstrating that 3D‐AAO nanostructures with optimized transverse channel distances can achieve remarkable radiative cooling performance. Diaz‐Lobo, et al.^[^ ^18^ ^]^ study showed that 3D‐AAO metamaterials on aluminum bulk with larger transverse channel distances exhibit high solar reflectance and high mid‐IR emissivity, acting as Bragg reflectors. The porosity is significantly higher than conventional AAO due to the periodically ordered transverse channels. Under direct sunlight, these structures achieved a maximum temperature reduction of 10.2 °C, establishing new benchmarks for all‐day passive radiative cooling performance based on porous alumina.

Complementing these radiative cooling capabilities, foundational work on tailoring thermal conductivity in 3D‐AAO templates has unlocked additional avenues for energy‐efficient thermal management. Studies have demonstrated that highly porous, interconnected 3D alumina networks can achieve significantly reduced thermal conductivity values of ≈0.5–0.8 W·m^−1^·K^−1^ compared to ≈1.5 W·m^−1^·K^−1^ for AAO templates for AAO templates (although this value of thermal conductivity of AAO has been shown to depend on the counter ions used during anodization and on the thermal history of the alumina templates^[^ ^19^, ^20^ ^]^), representing a ≈2–3‐fold reduction. This is important for example in thermoelectric applications when nanowires have very low thermal conductivity, lower than the AAO alumina templates. This reduction is attributed to enhanced phonon scattering within the 3D structure, making these networks ideal for thermal insulation, thermoelectric devices, and heat dissipation in microelectronics—thus bridging radiative and conductive thermal properties for multifunctional applications.^[^ ^20^ ^]^ As an example, the thermal conductivity values of 3D networks (0.5–0.8 W·m^−1^·K^−1^) show ≈3–4× reduction compared to bulk/polycrystalline Bi_2_Te_3_ (≈2.2 W·m^−1^·K^−1^ at 300K, depending on crystallographic orientation and microstructure).^[^ ^21^, ^22^ ^]^ In the recent literature, it has been demonstrated that modulating the refractive index of 3D alumina networks can merge the optical and the radiative cooling properties obtaining also dynamic and colorful radiative cooling applications, showing the versatility of these structures for thermal management while maintaining aesthetic appeal.^[^ ^18^, ^23^ ^]^

## 3 3D Nanonetworks as Functional Metamaterials: Properties by Design

Building on the photonic and thermal functionalities described in the previous section, the following section examines how the 3D interconnectivity of AAO templates enhances performance across magnetic, electronic, catalytic, energy storage, and sensing applications.

The full potential of 3D‐AAO templates will be unlocked by infiltrating their interconnected voids with functional materials as it has been done in the past for AAO templates. Techniques such as Atomic Layer Deposition (ALD),^[^ ^24^ ^]^ Electrodeposition,^[^ ^5^, ^25^ ^]^ Sol‐Gel Infiltration,^[^ ^26^ ^]^ Chemical Vapor Deposition (CVD),^[^ ^27^ ^]^ In Situ Polymerization,^[^ ^6^, ^28^ ^]^ melt‐infiltration,^[^ ^29^, ^30^ ^]^ to name a few, can also be used for the 3D‐AAO templates and will result in continuous 3D nanonetworks. These structures exhibit exceptional mechanical robustness and effectively scale up the unique properties of individual nanowires to the macroscopic level. The resulting samples can be studied and applied either with the nanonetwork embedded within the 3D‐AAO template or by selectively dissolving the template to yield an isolated free‐standing 3D nanonetwork (3D‐NN)—whose properties may differ from those of the infiltrated composite, as we will explore further.

### 3.1 Engineering Magnetic 3D Nanonetworks

**Figure 2:**
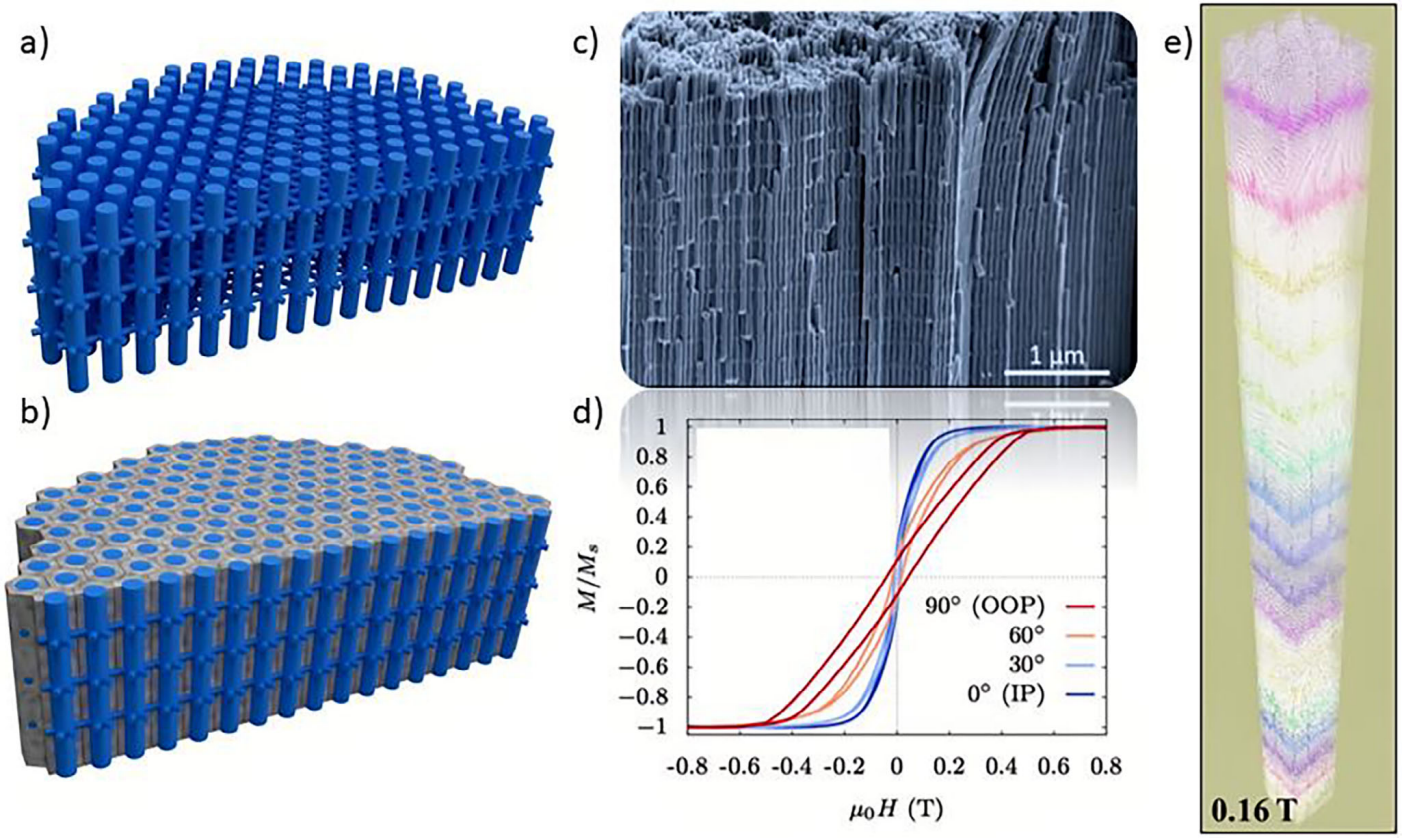
3D nanowire network design and magnetic properties. a) Schematic of a free‐standing 3D interconnected nanowire architecture after selective etching of AAO. b) Corresponding network embedded within a 3D anodic alumina oxide (3D‐AAO) template, highlighting the periodic and transverse interconnections. c) Cross‐sectional SEM image of a free‐standing Ni 3D nanowire network (scale bar: 1 µm) showing uniform vertical alignment and structural regularity. d) Angular‐dependent magnetic hysteresis loops reveal tunable magnetic anisotropy with progressive reorientation as a function of applied magnetic field angle. e) Micromagnetic simulation (MuMax) illustrating complex magnetization reversal pathways, including flux‐closure domain formations, governed by the network's geometric architecture. This geometry‐driven modulation of magnetic anisotropy and domain behavior demonstrates the powerful functional tailoring achievable in 3D nanomagnetic systems (Figure adapted from refs. [32, 33]).

Controlling magnetic anisotropy is of paramount importance for applications in data storage, sensing, and spintronics.^[^ ^31^ ^]^ By infiltrating 3D‐AAO templates with magnetic materials such as Nickel (Ni), Cobalt (Co), and its alloys (Ni_1‐x_Co_x_) it has been demonstrated that magnetic properties can be precisely controlled through architectural design. In a compelling demonstration of geometric control, it was shown that the magnetic easy‐axis of 3D Ni nanonetworks could be entirely reversed simply by tuning the interconnection distance,^[^ ^32^, ^33^ ^]^ **Figure** **2**. For large values of P (approaching the characteristics of isolated 1D nanowires), the easy‐axis is oriented out‐of‐plane (OOP), along the nanowire length, consistent with expectations due to shape anisotropy. However, as P is reduced (e.g., to ≈ 240 nm), strong magnetostatic interactions mediated by the transverse nanowires compel the easy‐axis to reorient to an in‐plane (IP) configuration. This architectural control over a fundamental magnetic property provides a powerful new design paradigm for 3D magnetic devices. A comprehensive review on the fabrication methods for 3D interconnected magnetic nanostructures, demonstrates how precise control over geometric parameters enables reversible tuning of magnetic anisotropy from out‐of‐plane to in‐plane orientations, thus providing a powerful design paradigm for next‐generation 3D magnetic devices and advancing understanding of their complex dynamic behaviors.^[^ ^31^ ^]^

Furthermore, the interconnected geometry introduces novel magnetization reversal behaviors. Utilizing a combination of First Order Reversal Curve (FORC) analysis (a powerful method for characterizing magnetic interactions and reversal processes) and micromagnetic simulations, researchers have discovered that reversal in dense 3D networks is driven by “highly localized magnetic states” and a previously unidentified “in‐plane magnetoelastic anisotropy” that originates from stress at the nanograin level. The transverse nanowires function as pinning sites for domain walls and promote unique reversal configurations, such as a “corkscrew‐like” arrangement of magnetization, as shown in Figure 2e.^[^ ^33^ ^]^ This is interesting because engineered pinning in cylindrical geometries enables deterministic nucleation, trapping, and release of domain walls and unlocks helical reversal modes not accessible in planar conduits. This study has unveiled the complex magnetization reversal process in 3D nickel nanowire networks, revealing how the interconnected architecture creates novel magnetic domain configurations and reversal pathways not observed in simpler 1D systems, underscoring the significance of 3D curvature and topology in tailoring domain‐wall energetics and motion.

Recent advances have revealed even more sophisticated magnetic behaviors in 3D nanonetworks. It has been recently demonstrated^[^ ^34^ ^]^ that magnetoelastic anisotropy drives localized magnetization reversal in 3D nanowire networks, providing new insights into the complex interplay between mechanical stress and magnetic behavior in these interconnected systems. Their work showed that the 3D architecture introduces unique stress distributions that fundamentally alter the magnetization reversal mechanisms compared to isolated nanowires. Additionally, it has been shown by micromagnetic simulations that tunable magnetic properties in interconnected permalloy nanowire networks can achieve specific magnetic functionalities. Micromagnetic simulations have revealed that interconnectivity in permalloy nanowire networks induces a nonmonotonic coercivity behavior depending on the horizontal nanowire position and diameter, such as 20 nm versus 50 nm wires. These architectural effects enable a coercivity enhancement of up to ≈ 20% compared to equivalent nonconnected nanowire arrays without changing the material composition. Additionally, while the normalized remanence remains relatively constant, it is consistently reduced by ≈ 10%–15% in the interconnected systems due to interwire coupling and bridge reversal, with larger horizontal diameters further lowering remanence. These quantified improvements demonstrate that 3D geometric tailoring of the nanowire network architecture can effectively optimize hysteresis, coercivity, and remanence for advanced magnetic device applications, providing a novel route to tune magnetic performance through structure rather than composition.^[^ ^35^ ^]^ These findings underscore the potential for geometrically tailored 3D networks in optimizing hysteresis, coercivity, and remanence for advanced magnetic devices.^[^ ^31^, ^32^, ^33^, ^34^ ^]^

The interconnected architecture of 3D‐AAO‐based magnetic nanonetworks enables precise modulation of the strong uniaxial shape anisotropy that typically governs their magnetic response, leading to distinctive micromagnetic states. This tunability unlocks their potential in diverse applications, including targeted drug delivery systems and magnetic hyperthermia therapies. Furthermore, the unique properties of these 3D nanonetworks hold promise for future advancements in high‐density data storage media, terahertz emission devices, and nanoelectronic components.^[^ ^31^, ^32^, ^33^, ^34^, ^36^, ^37^ ^]^

This geometric control, together with a comprehensive understanding of 3D magnetization phenomena, is pivotal for designing next‑generation spintronic devices that rely on precise domain‑wall control, including racetrack memories and domain‑wall logic, in which tuned pinning landscapes reduce stochasticity (engineered wall traps make switching more predictable) and improve energy efficiency (lower write/shift currents and fewer retries).

### 3.2 3D Nanonetworks for Energy Efficiency

**Figure 3:**
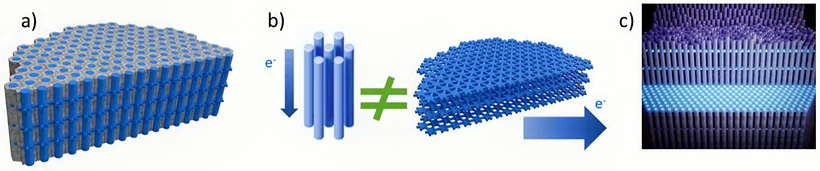
Directional and collective electron transport in 3D‐ordered nanowire networks. a) Schematic of a vertically aligned, periodically ordered Bi_2_Te_3_ nanowire architecture within a 3D‐AAO template. b) Comparison of electron transport in isolated 1D nanowires (left), where connectivity is limited, versus the enhanced electrical connectivity and charge transport in the interconnected planes of the network (right). c) Visualization of electron flow throughout the interconnected nanonetwork, highlighting how advanced 3D architectures substantially optimize carrier mobility and electronic performance—key for energy conversion and thermoelectric applications. (ref. [22]).

Thermoelectric devices (TEGs) convert heat directly into electricity, offer a promising avenue for waste heat recovery. Their efficiency is quantified by the dimensionless figure of merit, *zT* = (*S^2^·σ*/*k*)T, where *S* represents the Seebeck coefficient, *σ* denotes the electrical conductivity, and *k* signifies the thermal conductivity. The primary challenge lies in reducing *k* without concurrently degrading the power factor (*S^2^·σ*). By synthesizing 3D interconnected networks of bismuth telluride (Bi_2_Te_3_), a well‐established thermoelectric material, a substantial *z*T improvement at room temperature versus 1D/film counterparts prepared by the same electrodeposition route has been observed, driven by *k* reduction and enhanced *S*.^[^ ^22^, ^38^ ^]^ The synthesis and optimization of these 3D Bi_2_Te_3_ networks have been detailed, providing a robust foundation for their enhanced thermoelectric performance.^[^ ^11^ ^]^ The 3D network introduces exceptional phonon scattering, reducing *k* to values as low as 0.5 W·m^−1^·K^−1^. This phenomenon was attributed to a “heat viscosity concept”, where the term describes collective phonon transport resistance at nanoscale junctions. This has been experimentally verified through measurements showing 40%–60% reduction in thermal conductivity at junction interfaces compared to continuous nanowires. This phenomenon results from enhanced phonon‐boundary scattering and phonon localization effects specific to the 3D interconnected architecture.^[^ ^22^ ^]^ Although another suitable explanation is that the 3D‐AAO architectures have an increased number of interfaces for phonon scattering compared to the AAO nanowires counterpart,^[^ ^39^ ^]^ explaining an extra reduction of the thermal conductivity.Complementary low‑temperature transport shows localization and directionality of surface conduction along orthogonal paths in ordered 3D Bi_2_Te_3_ metamaterials, providing a microscopic picture of geometry‑tunable electronic pathways coexisting with junction‑dominated phonon suppression, as shown in **Figure** **3**.^[^ ^38^, ^39^ ^]^

These geometry‑induced effects generalize to sustainable alloys: 3D‐CuNi interconnected nanonetworks have revealed an ≈5x higher *z*T than the bulk material, attributable to ultralow lattice thermal conductivity, attained through a dual nanostructuring strategy combining saccharine‐induced crystallite size reduction and 3D‐AAO templating. The lattice thermal conductivity was further reduced, underscoring the crucial role of the 3D nanonetwork in phonon transport for inorganic materials.^[^ ^40^ ^]^ It is also shown in that work that the observed hierarchy— films → 1D nanowires → modulated nanowires → fully interconnected 3D networks— monotonically lowers *k* through enhanced boundary and junction scattering, with the largest reductions in the 3D morphology similarly as observed for 3D Bi_2_Te_3_ nanonetworks.

For organic semiconductors such as PEDOT, the 3D network approach may not be fully adequate. due to challenges in achieving uniform pore infiltration, stable doping within 3D‐AAO templates, or simply the way the polymer chains are within the 3D‐AAO structure. These issues can hinder optimal decoupling of electrical and thermal transport—potentially arising from uneven electropolymerization kinetics, incomplete filling of interconnected voids, or dopant instability in confined geometries ‐ and collectively reduce the potential for phonon scattering and high *zT* values.^[^ ^41^, ^42^ ^]^

To address these limitations, 3D PEDOT nanowire networks fabricated via electropolymerization in 3D alumina templates have been explored, yielding free‐standing structures with vertical nanowires (≈40 nm diameter) and transverse connections (≈120 nm spacing), an electrical conductivity of 43 ± 5 S cm^−1^, and a Seebeck coefficient of 28 ± 2 µV K^−1^,—highlighting enhanced surface area for applications like supercapacitors and flexible energy devices.^[^ ^42^ ^]^

While optimized PEDOT films can achieve a power factor,^[^ ^42^ ^]^ future optimization for organic 3D thermoelectrics may require improved infiltration protocols, advanced doping schemes, improve the polymer chain orientation, and composite formation to decouple electrical and thermal transport.

Recent breakthroughs in 3D‐AAO templating have enabled the fabrication of electrochemical double‐layer capacitors (EDLCs) by constructing compactly arranged 3D carbon tube (3D‐CACT) nanoarrays directly within anodic aluminum oxide templates. By systematically tuning the vertical pore diameter (70–250 nm) and interpore distance (110–450 nm) via controlled anodization protocols, these 3D‐CACT electrodes achieve a specific surface area of 253.0 m^2^ g^−1^. Consequently, sandwich‐type EDLCs based on 3D‐CACT exhibit an areal capacitance of 3.23 mF cm^−^^2^ at 120 Hz alongside a phase angle of –80.2°, indicative of a superior fast‐frequency response suitable for AC line‐filtering applications. This precise architectural control breaks the conventional trade‐off between energy density and frequency response in miniaturized capacitors, presenting a scalable and straightforward route for high‐performance microelectronic filtering components.^[^ ^43^ ^]^

Complementing the high‐density approach, 3D carbon tube grids (3D‐CTGs) fabricated using 3D‐AAO templates have demostrated high‐frequency line‐filtering performance with phase angles approaching – 90° at operational frequencies, and structural advantages stemming from a well‐organized, integrated 3D architecture that provides fast ion migration channels and excellent electronic conductivity. This unique 3D‐AAO‐enabled architecture provides efficient ion transport pathways while maintaining structural stability, representing an advanced in high‐power line filtering capacitor design.^[^ ^44^ ^]^

A major advancement in sustainable energy harvesting using 3D‐AAO templates has been achieved through the development of composite triboelectric nanogenerators (TENGs) based on polylactic acid (PLA) infiltrated within the 3D‐AAO porous network. These nanoengineered composites serve as the active triboelectric layer, efficiently converting mechanical energy into electrical output. The resulting TENGs^[^ ^45^ ^]^ demonstrate a power density of 1.08 W per m^2^ under actuation. This power output is relevant for the Internet of Things (IoT) applications as demostrated of a wireless Bluetooth operation via rectification and capacitor charging, highlighting a scalable path to eco friendly, self powered wearable and sensor nodes. The 3D AAO framework enhances effective permittivity and mechanical stability while preventing filler agglomeration, and free standing 3D PLA nanonetworks obtained by template removal further show strong gains over bulk PLA, illustrating how ordered 3D nanostructuring improves triboelectric performance alongside biodegradability options.

### 3.3 3D Nanonetworks for Energy Storage

The application of 3D‐AAO templating to battery electrode fabrication has yielded transformative results in both Li–O_2_ and hybrid Li‐ion battery systems, demonstrating the versatility of 3D architectures in energy storage applications. Carbon‐coated 3D Ni nanomesh electrodes fabricated via AAO templating deliver ≈100× higher charge‐footprint capacity versus planar glassy carbon; thin MnO_2_ films on the nanomesh exhibit hybrid Li‐ion/Li–O_2_ behavior. These electrodes exhibit an exceptional surface area‐to‐footprint ratio of 90 cm^2^:1 cm^2^, enabling unprecedented active material loading per unit area. The 3 µm‐thick nanomesh features uniformly distributed nanowires (40 nm diameter) with an inter‐wire spacing of 60 nm inter‐wire spacing, creating an interconnected network that promotes thin Li_2_O_2_ layer formation (8–10 nm) with high reversibility (87.5% faradaic efficiency). As a result, the carbon‐coated 3D Ni nanomesh demonstrates an outstanding areal capacity, significantly surpassing planar glassy carbon electrodes and overcoming the limitations of thick Li_2_O_2_ deposits that plague conventional Li–O_2_ systems.^[^ ^46^ ^]^

Building on the 3D nanomesh platform, amorphous MnO_2_ coatings deposited via liquid atomic layer deposition have demonstrated novel hybrid Li‐ion/Li–O_2_ behavior. Upon cycling under O_2_, the MnO_2_‐coated nanomesh undergoes partial lithiation to LiMnO_2_, which acts as a redox mediator: O_2_ exposure reactivates electrochemically deactivated LiMnO_2_, enabling sustained cycling performance. Over 10 cycles, charge densities gradually increase for both reduction and oxidation. The dual functionality of the MnO_2_ layer—combining Li‐ion intercalation and Li–O_2_ formation mechanisms—provides superior energy storage capability and addresses the conventional limitations of both pure MnO_2_ electrodes and Li–O_2_ systems, showcasing the unique opportunities enabled by 3D‐AAO‐templated architectures.^[^ ^47^ ^]^

Extending 3D‐AAO's utility in energy storage, recent work has fabricated 3D multi‐layer carbon tube (3D‐MLCT) frameworks via CVD and ALD within 3D‐AAO templates, achieving tunable inter‐layer spacings and high areal capacitance with fast frequency response for AC line‐filtering capacitors. This integrated tube‐in‐tube design enhances ion diffusion and surface area, leveraging the 3D interconnectivity to achieve significantly higher energy density and cycling stability than non‐interconnected structures, while enabling efficient filtering of pulse voltage signals from triboelectric nanogenerators (TENGs). This highlights 3D‐AAO's potential for scalable, different high‐performance energy devices.^[^ ^48^ ^]^

The use of 3D anodized aluminum oxide (AAO) templates to fabricate interconnected vanadium pentoxide (V_2_O_5_) electrodes offers significant advantages over conventional 1D nanowires or bulk materials. By introducing controlled interconnections between the pores in the AAO scaffold, a bicontinuous porous network is created, which enhances both electronic and ionic transport pathways within the electrode. This architecture increases accessible surface area and facilitates electrolyte infiltration, reducing diffusion lengths and improving rate capability.^[^ ^49^, ^50^ ^]^ However, it is important to note that the performance of V_2_O_5_ strongly depends on its intrinsic electronic conductivity and the lithiation depth. Literature reports show that while interconnected structures can improve capacity retention compared to planar electrodes under moderate cycling conditions, excessive lithiation (>1 Li per V_2_O_5_ unit) may lead to structural stress and hinder rate performance.^[^ ^49^, ^50^ ^]^ For example, Kim et al.^[^ ^49^ ^]^ observed improved cycling stability in interconnected mesoporous V_2_O_5_ networks, but the retention values vary with architecture and test protocol; thus, generalized performance metrics should be interpreted cautiously by now and supported by furhter experimental work.

### 3.4 Catalytic Applications of 3D‐AAO Networks

Zankowski et al. have demonstrated how high porosity can be combined with high surface area in flexible interconnected nanowire meshes through rational template design, with applications extending from hydrogen generation to beyond. Their work showed that the 3D architecture provides optimal mass transport properties while maintaining high catalytic activity. Specifically, they developed electrochemical methods—combining cyclic voltammetry (CV) and electrochemical impedance spectroscopy (EIS)—for determining the surface area of thin films of interconnected nickel nanowires, and gravimetric electrodeposition techniques for porosity quantification, validated by gas–adsorption measurements where applicable, providing an interesting characterization tools for the field.^[^ ^51^ ^]^ The field has also seen advances in 3D energy storage applications, particularly in microsupercapacitors, where interconnected nanowire networks with interdigitated electrode designs have demonstrated enhanced areal energy and power capabilities. The interconnected geometry, when properly engineered with controlled nanowire diameter and spacing, provides superior ion and electron transport pathways compared to conventional planar designs and random microporous structures.^[^ ^52^ ^]^

### 3.5 Designing Advanced Polymeric 3D Metamaterials

**Figure 4:**
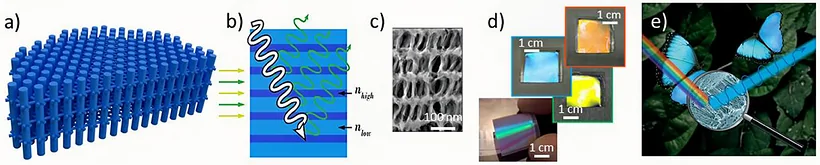
Bioinspired polymeric photonic architectures with tunable structural color. a) 3D schematic of a free‐standing, periodic nanoporous polymer multilayer, mimicking the hierarchical design of butterfly wing scales. b) Illustration of Bragg reflector behavior arising from the alternating refractive index layers within the nanonetwork. c) SEM image (scale bar: 100 nm) displaying the ordered, hierarchical pore arrangement responsible for the structure's photonic properties. d) Photographs highlighting the dynamically tunable, vivid structural coloration, iridiscence, and mechanical flexibility of these pigment‐free polymeric networks; natural butterfly wing coloration is provided for direct comparison. Visual demonstration of the analogous optical phenomena observed in blue morpho butterflies or Jeweled Beetle with vibrant coloration and iridiscent behaviour, showcasing the biomimetic nature of the synthetic networks (adapted from ref. [9]). And e) These Bragg‐based nanonetworks enable programmable, environmentally sustainable, and flexible color generation through precise nanoscale structural engineering (adapted from ref. [9]).

The 3D‐AAO platform is also highly efficacious for the creation of functional polymeric nanostructures with diverse applications in photonics and energy harvesting. By infiltrating 3D‐AAO templates with a biodegradable polymer, such as polylactic acid (PLA), and subsequently dissolving the template, it becomes feasible to produce a free‐standing, single‐material polymer DBR.^[^ ^53^ ^]^ This anti‐replication process yields the “negative” of the 3D‐AAO template, a periodic network of air voids within the PLA, thereby generating the requisite refractive index contrast. These all‐polymer DBRs achieve exceptionally high reflectance (>95%) with merely ≈ 20 repeating layers, thereby overcoming a significant limitation of conventional polymeric photonic crystals, see **Figure** **4**. Their inherent flexibility and biodegradability render them ideal for sustainable optical components in flexible displays or biomedical sensors. Additionally, cost‐effective, flexible, hydrophobic, and tunable structural color polymeric Bragg reflector metastructures have been fabricated through the melt infiltration of polyethylene into 3D‐AAO templates, demonstrating chameleonic behavior by altering color when exposed to solvents with varying refractive indices.^[^ ^9^ ^]^ Building on this, infiltrated 3D‐AAO with varying polyethylene (PE) densities, revealing that chain branching in low density polyethylene (LDPE) enhances macroscopic integrity while enabling tunable photonic responses via refractive index modulation.^[^ ^54^ ^]^

### 3.6 Sensing and Biomedical Applications

As photonic crystals, modulated‐AAO stacks and 3D porous AAO can operate as highly sensitive label‑free optical biosensors. Their large internal surface area offers abundant functionalization and analyte‑binding sites, which boost sensitivity relative to simple 1D pore arrays. In the visible–near‑IR, AAO photonic crystals with ≈300–400 nm periods exhibit well‑defined stop bands whose spectral positions shift with the period, effective refractive index, and pore filling—enabling refractive‑index sensing and spectral engineering.^[^ ^55^ ^]^ The 3D‐AAO platform has enabled advancements in plasmonics, with studies on plasmon resonances in 3D nanowire networks of topological insulators and metals like Ni.^[^ ^56^ ^]^

Beyond sensing, 3D‑AAO templating enables electrically continuous Bi_2_Te_3_ nanowire networks that combine plasmonic response (NIR–MIR) with high thermoelectric performance—without adding noble‑metal components. Topological insulators (TI) chalcogenides and their nanostructures support plasmon‑like resonances and strong photothermal conversion; illumination can thus produce localized heating within the network. Meanwhile, the 3D nanowire architecture intrinsically suppresses lattice thermal conductivity (via enhanced phonon scattering as shown before^[^ ^22^ ^]^), thereby improving the thermoelectric figure of merit compared with comparable 1D arrays and thin films. This single‑material, large‑area process reduces metal/TI interfacial losses, simplifies fabrication, and is compatible with scalable manufacturing. These geometry‑driven photothermal/thermoelectric synergies open opportunities in wearable thermoelectric generators, solar‑assisted energy harvesting, and localized thermal management.^[^ ^38^, ^56^, ^57^, ^58^, ^59^ ^]^

In principle, Dirac plasmons on topological insulator surfaces (such as Bi_2_Te_3_) or in doped graphene can can be optimized by engineered 1) surface‐state carrier density, 2) network periodicity, and 3) dielectric environment. First, tuning the Fermi level via chemical doping or charge‑transfer overlayers increases the 2D surface‑state carrier density, raising the Dirac‑plasmon frequency according to ω_p_ ∝ n^1/4^√q (at fixed q), which can move the resonance toward the THz–mid‐IR range. Chemical doping or charge‐transfer overlayers that raise n_sur_𝒻 increase ω_p_. and can move the plasmon into the desired THz–mid‐IR window. Electrostatic gating is powerful in planar films or single nanowires but is more challenging to apply uniformly in 3D networks.

Second, to place a photonic band edge at λ ≈ 6 µm in periodic 3D architectures (such as AAO‐templated nanowire networks), the AAO periodicity should be micrometre‑scale (e.g., a 1D Bragg period Λ≈ λ / 2n_eff_) ≈ 2 µm for n_eff_ ≈ 1.5; 2D lattices typically require a ≈ 1–3 µm, depending on geometry and fill factor). Using a ≈ 300–350 nm would instead produce visible/near‑IR band edges. Aligning the photonic band edge with the Dirac‑plasmon frequency maximizes hybridization strength for engineered 3D nanostructured networks.

Third, applying a low‑loss conformal cladding (e.g., ALD Al_2_O_3_) modifies ε_env_, passivates surfaces, and can improve confinement and reduce scattering losses, which is helpful for increasing quality factor. At room temperature in the mid‑IR, Q‑factors of order tens are realistic starting points; Q > 100 generally demands exceptional materials and/or reduced temperature. Target strong coupling (anti‑crossing) by maximizing spectral overlap and minimizing damping; treat Rabi splitting of tens of meV as a design goal pending system‑specific validation.^[^ ^60^, ^61^ ^]^

As an example of future development in the fields, It is clear that this intricate 3D‐AAO pore geometry is eminently suitable for the creation of sophisticated drug delivery systems or even tissue engineering.There is a work on flexible 3D nanonetwork silica films in which it has been developed a polymer‐free drug‐eluting stent platforms, providing an effective way of drug release that significantly reduced tissue hyperplasia in an affected rat esophagus. This work demonstrates the biocompatibility and functionality of 3D nanonetworks in biomedical applications.^[^ ^62^ ^]^

## 4 Applications, Scalability, and Industrial Outlook

For any nanotechnology to achieve substantial real‐world impact, its scalability and cost‐effectiveness are paramount. Significant advancements have been made in transitioning 3D‐AAO from a laboratory curiosity to an industrially viable platform, including streamlined anodization techniques that enable large‐scale production of interconnected nanoporous templates for real world applications. The technology is advancing toward continuous production models tailored for 3D‐AAO, such as hybrid pulse anodization (HPA) adaptations that support roll‐to‐roll processing on flexible aluminum foils, enabling large‐area films with interconnected porosity for emerging applications including radiative cooling and filtration. These developments indicate a trend toward industrial‐scale fabrication, with potential for integration into existing industrial aluminum processing lines.

Current scalability challenges in large area fabrication of 3D‐AAO in the lab include maintaining uniform current distribution over large areas (>10 cm^2^), controlling temperature variations during extended anodization (>24 h), and ensuring consistent electrolyte composition. Although these issues are routinely managed in industrial anodizing line, as large‐area anodized pieces are being processed industrially worldwide every day.^[^ ^63^, ^64^ ^]^ Also, historically, the need for ultra‐high‐purity aluminum (≥99.9999%) has driven material costs, but recent innovations using less pure aluminium as starting material can transform this landscape. By employing aeronautic‐grade^[^ ^9^ ^]^ or even commercial‐grade^[^ ^12^ ^]^ aluminum, yields to high‐quality, interconnected 3D‐AAO while reducing raw‐material expenses by over 90%. Studies^[^ ^12^ ^]^ comparing 99.5% versus 99.9% aluminum purity show that lower purity results in 15%–20% increased surface roughness and 10%–15% variation in pore diameter uniformity, while maintaining structural integrity for most applications. Despite these variations, the fundamental 3D‐AAO architecture and functionality remain intact, making the technology accessible for cost‐effective industrial applications using lower‐grade aluminum substrates, if that is a requierement.

Additionally, if ordering and homogeneity in pore shape is not a critical issue for a specific application, 3D‐AAO can be obtained at near room‐temperature operation—thereby eliminating the cost of cooling and achieving substantial reductions in chiller load.^[^ ^65^ ^]^ Coupled with roll‐to‐roll anodization processes and template reuse, these advances pave the way for economically viable, large‐area production of 3D‐AAO for energy, photonic, and other advanced device applications.

Roll‐to‐roll (R2R) anodization of nanoporous alumina offers a compelling route to continuous, large‐area production of AAO templates, yet it remains constrained by several technical hurdles. First, the need to dissipate Joule heating during extended anodization demands active cooling systems—typically maintaining the bath at ≈10 °C—which increases both capital and operational costs. Second, achieving uniform current distribution across meter‐scale foil widths requires precisely engineered electrode geometries and real‐time current monitoring; without these measures, pore size and oxide thickness can vary by up to 15%, undermining template reproducibility. Third, electrolyte aging due to Al^3^⁺ accumulation necessitates frequent bath replacement or continuous filtration, adding complexity to process infrastructure. Additionally, low anodization rates (≈0.2 µm·min^−1^) limit throughput, often requiring >10 h to attain useful oxide thicknesses, which constrains industrial scalability. Nonetheless, R2R anodization presents distinct advantages: it enables seamless integration with existing aluminum foil processing lines, eliminates batch‐to‐batch variability inherent in static cells. If the nanopores ordering and shape it is not an issue for the application, operating anodization at or near room temperature substantially reduces cooling requirements compared to conventional processes that maintain electrolyte baths at ≈10 °C or lower.^[^ ^12^ ^]^ This approach, combined R2R, Hybrid Pulse Anodization (HPA, which reduce cooling needs 20–30 °C^[^ ^66^ ^]^), and it is compatible with commercial‐grade aluminum, can significantly lower energy consumption by minimizing refrigeration demand. While exact savings depend on bath size, current density, and process duration, published analyses indicate meaningful reductions in operational energy rather than a fixed percentage value.^[^ ^65^, ^66^, ^67^ ^]^

**Figure 5:**
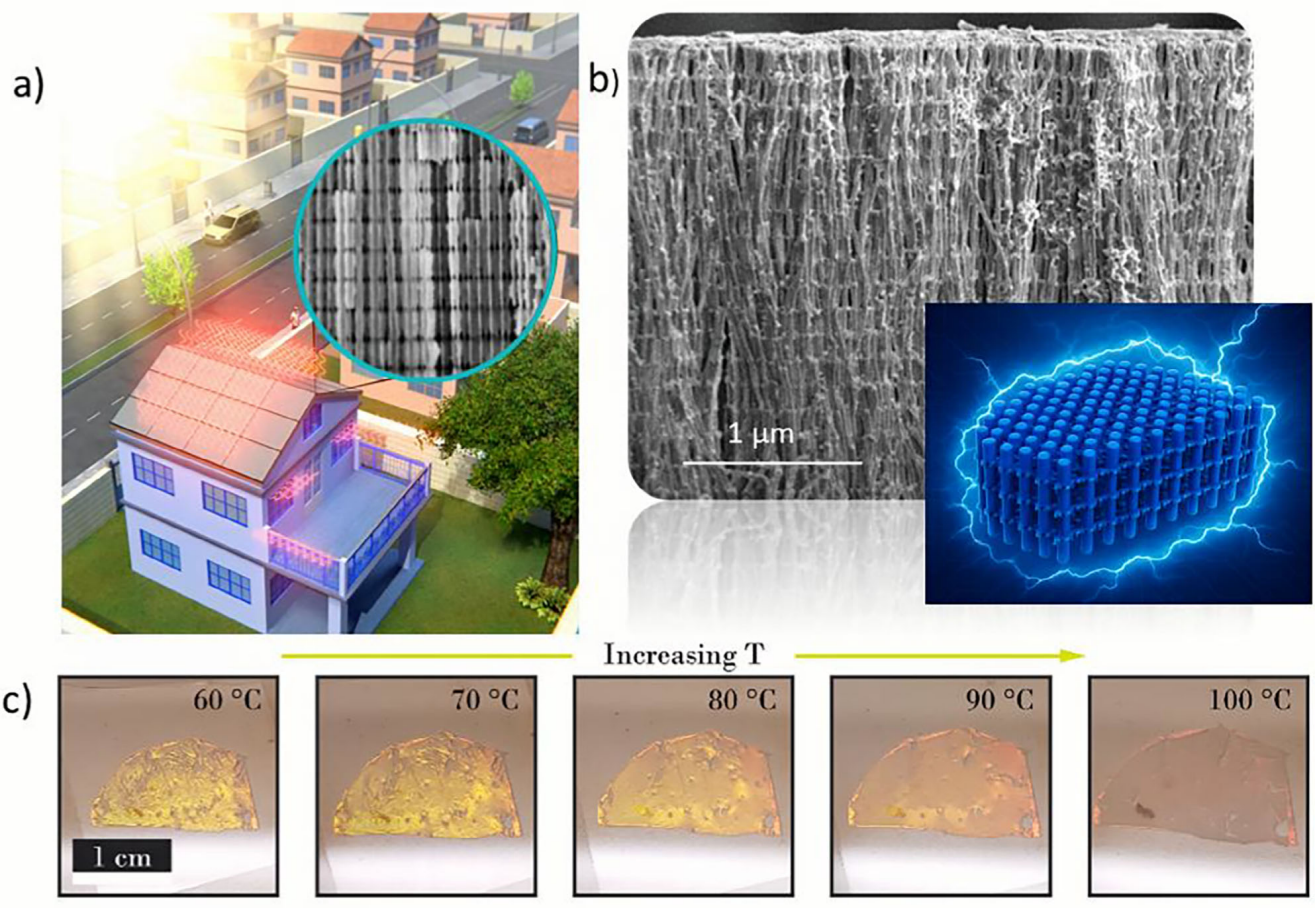
a) Schematic illustration of a 3D anodic aluminum oxide (3D‐AAO) nanonetwork applied as a radiative cooling layer on a building surface. The inset highlights the interconnected nanoporous architecture that enhances solar reflectivity and mid‐infrared emissivity, enabling effective sub‐ambient passive cooling (adapted from ref. [18]). b) Electron microscopy image (scale bar: 1 µm) of a free‐standing 3D polymeric nanonetwork poly(lactic acid) (PLA) employed as a triboelectric nanogenerator (TENG). The inset conceptually illustrates energy harvesting by the 3D polymer nanonetwork. The hierarchical structure increases contact area and compliance, enhancing mechanical‑to‑electrical energy conversion (adapted from ref. [45]). c) Visually readable temperature indicator based on a free‑standing polymeric nanonetwork. Above ≈100 °C the network collapses irreversibly, enabling naked‑eye detection. The collapse threshold is tunable via polymer selection, making it suitable for applications in food safety and smart packaging.

Several 3D‐AAO applications show near‐term commercial viability including passive radiative cooling for building applications, triboelectric nanogenerators for wearable electronics, high‐performance supercapacitors for energy storage systems, or temperature sensors for food industry. The combination of scalable fabrication, cost‐effectiveness, and demonstrated performance makes these applications particularly promising for industrial implementation, **Figure** **5**.

## 5 Conclusion and Future Perspectives

The trajectory from the initial ordered 3D‐AAO nanoarchitecture in 2014 to the functional, high‐performance metamaterials observed today exemplifies a remarkable decade of scientific advancement. By transforming anodic alumina from a template for preparing 1D nanowires into a programmable 3D architectural metamaterial platform, researchers have gained the ability to engineer material properties in an entirely novel manner. Achievements such as the potential for tuning magnetic properties through architectural design through interconnection tuning, enhancement of thermoelectric performance through plasmonic heating and engineered phonon scattering mechanisms, and the realization of full‐gamut structural colors in hybrid photonic‐plasmonic systems unequivocally demonstrate the efficacy of this approach. Recent breakthroughs in radiative cooling, where 3D‐AAO structures achieve temperature reductions achieving up to ∼10 °C below ambient outdoors under full sun (in Madrid Spain, summer; aluminum substrate). These results showcase its potential for energy‐efficient thermal management. The adoption of this technology by the global research community affirms the fundamental importance of the 3D interconnected architecture.

With clearly defined pathways toward low‐cost, scalable production—such as single‐step pulsed anodization reducing fabrication time to under 24 h and enabling use of low‐purity aluminum—3D‐AAO is well‐positioned for significant commercial impact. The advancements in 3D‐AAO fabrication have not only redefined a scientific domain but have also sparked a wave of innovations across multiple disciplines, warranting further exploration of this versatile platform.

It is clear that this intricate 3D pore geometry is eminently suitable for the creation of sophisticated drug delivery systems or even tissue engineering. As an examples of future development, flexible 3D nanonetwork silica films have been developed as polymer‐free drug‐eluting stent platforms, providing an effective way of drug release that significantly reduced tissue hyperplasia in an affected rat esophagus.

Incorporating 3D AAO templates with tunable pore diameters in the 100–500 nm range—though not addressed in the current body of work—would be of high interest. Such control over pore size could enable enhanced phonon scattering for ultra‐low thermal conductivity in thermoelectric generators, improved optical stop‐band tuning for mid‐infrared radiative cooling coatings, and tailored mass‐transport pathways in microfluidic or catalytic reactors. Future efforts to establish robust protocols for oxide growth under oxalic and phosphoric electrolytes would therefore open new avenues for multifunctional 3D nanoporous metamaterials.

Development of 3D AAO scaffolds featuring precisely defined pore diameters between 100  and 500 nm remains an exciting challenge. Achieving this degree of architectural control—by varying electrolyte composition (e.g., oxalic vs phosphoric acids), current density, and temperature—could: 1) Enhance thermoelectric performance via geometry‐driven phonon blockade; 2) Extend photonic stop‐band tunability for adaptive radiative cooling surfaces; and 3) Optimize fluidic and ionic transport in 3D microreactors and sensor platforms. Pursuing these investigations will significantly enrich the functional scope of 3D nanoporous alumina in energy conversion, thermal management, and catalysis.

Looking forward, the field presents numerous opportunities for further exploration. Future research endeavors are likely to focus on creating even more intricate, hierarchical structures, potentially integrating multiple materials or functionalities within a single network. The convergence of 3D‐AAO fabrication techniques with artificial intelligence and machine learning could facilitate AI‐driven design of anodization protocols, thereby enabling the realization of previously unattainable architectures. Integration with 2D materials like graphene and MXenes shows promise for quantum applications, with challenges including interface adhesion, charge transfer efficiency, and maintaining 2D material properties in confined geometries. Recent preliminary work suggests potential for novel hybrid systems with enhanced functionality. With AI‐assisted design and integration with emerging quantum and graphene‐based materials, 3D‐AAO architectures stand poised to enable breakthroughs in sustainable energy, healthcare, and telecommunications, bridging fundamental materials science with broad societal impact.

## Conflict of Interest

The authors declare no conflict of interest.
